# Association of TNF-R1 with Exercise Capacity in Asymptomatic Hypertensive Heart Disease—Mediating Role of Left Ventricular Diastolic Function Deterioration

**DOI:** 10.3390/jcm14155391

**Published:** 2025-07-31

**Authors:** Anna Teresa Gozdzik, Marta Obremska

**Affiliations:** Division of Cardiovascular Imaging, Institute of Heart Diseases, Faculty of Medicine, Wroclaw Medical University, 50-556 Wroclaw, Poland; marta.obremska@umw.edu.pl

**Keywords:** hypertensive heart disease, TNF receptor 1, exercise capacity

## Abstract

**Background:** TNF receptor 1 (TNF-R1) mediates the proinflammatory and proapoptotic effects of TNF-alpha, with its soluble form predicting incident heart failure (HF). While there is evidence linking TNF pathway activation to cardiac dysfunction, the mechanisms involved remain unclear. This study aimed to investigate the association between TNF-R1, exercise capacity, and cardiac function in asymptomatic patients with hypertensive heart disease (HHD). **Methods:** We enrolled 80 patients (mean age 55 ± 12 years) with HHD and no clinical symptoms of HF (stages A and B). Echocardiography, including tissue Doppler and left atrial and left ventricular (LV) strain assessment, was performed at rest. Peripheral venous blood samples were collected to measure serum TNF-R1 concentration. **Results:** The study population was divided into two subsets based on the median exercise capacity (peak VO_2_) value. Patients with higher VO_2_ had lower serum TNF-R1 concentration and higher early peak mitral annular velocity (e’) and peak atrial longitudinal strain (PALS). After adjusting for other covariates, multivariable regression analysis identified TNF-R1 as an independent determinant of peak VO_2_. Mediation analysis revealed that the relationship between TNF-R1 and peak VO_2_ was mediated by LV diastolic function (PALS or e’), with a decrease in the beta coefficient after including mediator variables from 0.37 (*p* < 0.001) to 0.30 (*p* < 0.006) and 0.31 (*p* = 0.004), respectively. **Conclusions:** In patients with HHD, higher TNF-R1 levels are associated with lower exercise capacity, which may be mediated by impaired LV diastolic function. These findings might suggest a role of TNF signalling in early HF development, justifying further studies to evaluate TNF-R1 as a biomarker for risk of HF progression.

## 1. Introduction

Hypertensive heart disease (HHD) is a frequent precursor to the development of HFpEF (heart failure with preserved ejection fraction), a heart failure (HF) category with quickly increasing prevalence that currently accounts for nearly 50% of all HF cases [1,2]. Reduced exercise capacity develops gradually in HHD and precedes the onset of overt HF. The reasons for clinical deterioration include progressive myocardial morpho-functional derangements, such as left ventricular (LV) hypertrophy and diastolic and systolic dysfunction, all of which lead to the elevation of LV filling pressure and dyspnoea. The mechanisms responsible for this process are poorly recognized, which poses problems for adequately implementing preventive measures.

Cytokines play diverse roles in activating vascular cells and regulating the immune system in hypertension, both systemically and locally. This contribution can be seen even in the early stages of hypertension, with increased levels of proinflammatory cytokines, such as Tumour Necrosis Factor-alpha (TNF alpha). The majority of deleterious effects of TNF are mediated by the receptor R1 (TNF-R1), the activation of which stimulates proinflammatory and apoptotic pathways and aggravates cardiac remodelling and hypertrophy [3]. Data from experimental models of pressure overload suggest the active role of TNF-R1 signalling in the development of hypertensive cardiomyopathy. In clinical studies, a soluble form of TNF-R1 emerged as a strong independent predictor of HF severity, being associated with an increased risk for HFpEF and, to a lesser degree, HFrEF (heart failure with reduced ejection fraction) [4]. However, there is a paucity of data linking TNF R1 with exercise capacity in the preclinical stage of HF, which might shed light on the pathophysiology of the early phase of the development of functional limitations in HHD. Moreover, the detailed mechanisms behind the detrimental impact of TNF-R-1 signalling on the cardiovascular system, especially in less advanced stages of the HF cascade, are not entirely clear, and their recognition might be supportive in the diagnostic process identifying individuals at risk.

In view of this, we sought to investigate the association of TNF-R1 with exercise capacity, and cardiac function in asymptomatic patients with HHD. We hypothesize that higher TNF-RI may parallel reduced exercise capacity, with impaired cardiac function potentially contributing to this relationship.

## 2. Materials and Methods

### 2.1. Study Design

The study involved patients diagnosed with HHD, who exhibited no clinical signs or symptoms of HF, classified as stage A and B, and had adequately controlled blood pressure (BP), defined as office measurements below 140/90 mmHg according to current guidelines [5,6]. This was a prospective study, and participants were recruited consecutively from outpatient clinics at a tertiary care centre between 2019 and 2023.

Exclusion criteria for this study were absence of stable sinus rhythm, HF with left ventricular ejection fraction (LVEF) below 50%, history of coronary artery disease, presence of more than mild valvular heart disease, primary myocardial diseases, chronic obstructive pulmonary disease, positive exercise stress tests indicating myocardial ischemia, 24 h ambulatory blood pressure monitoring (ABPM) average results being equal to or higher than 155/90 mmHg at night, or equal to or higher than 165/105 mmHg during the day, and a hypotensive response to exercise, defined as a drop in systolic BP below resting levels or an initial increase in systolic BP followed by a decline of 20 mmHg or more. Following an initial screening which involved the analysis of medical documentation, an interview, and a physical examination, 80 patients were found to meet the criteria for participation in the study.

The study was carried out in compliance with the Declaration of Helsinki and was approved by the ethics committee (approval no. KB-482/2019). All participants provided written informed consent.

### 2.2. Study Protocol

Participants were examined during two separate visits. Prior to each visit, they were instructed to refrain from smoking, consuming caffeine or alcohol, and exercise for a minimum of three hours before their appointment. During the first visit, participants underwent a physical examination, standard anthropometric measurements, and automated office BP measurement while seated at rest. Two readings were performed, taken one minute apart, following a five-minute rest period on the arm with the higher BP readings. Additionally, 24 h ABPM was conducted.

The second visit took place the following day, during which participants returned to discuss the ABPM results. At this visit, blood samples were collected for laboratory analyses, followed by a resting echocardiographic examination and cardiopulmonary exercise testing.

### 2.3. Blood Assay

Peripheral venous blood samples were drawn during the second visit, between 8:00 a.m. and 9:00 a.m., following 30 min of rest in the supine position. Blood was collected for the measurement of hemoglobin, estimated glomerular filtration rate (eGFR), N-terminal pro–B-type natriuretic peptide (NT-proBNP), and soluble tumour necrosis factor receptor 1 (sTNF-R1). hemoglobin concentration was assessed using an automated hematology analyser. The eGFR value was calculated based on serum creatinine levels, age, and sex. NT-proBNP levels were measured using an ELISA assay.

For the determination of sTNF-R1, blood was drawn into serum-separating tubes, which were then centrifuged at 2000× *g* for 10 min at 4 °C. The resulting serum was stored at −80 °C until analysis. All samples underwent a single freeze–thaw cycle. Prior to analysis, samples were diluted 1:10 with the kit diluent and assayed for TNF-R1 concentration using a quantitative sandwich enzyme immunoassay (Quantikine^®^ ELISA Kit, R&D Systems Inc., Minneapolis, MN, USA), following the manufacturer’s instructions.

### 2.4. Echocardiography

Conventional and speckle tracking echocardiography was performed using standard equipment (Vivid E9, General Electric Medical Systems, Milwaukee, WI, USA) with phased array 2.5 MHz multifrequency transducers. Images were saved in digital format on a secure server for further offline analysis (EchoPAC, v. 204, GE Medical Systems). Cardiac dimensions and wall thicknesses were measured according to standard recommendations [7]. LA volumes were evaluated by the biplane Simpson method and indexed by body surface area. LV inflow parameters, including peak early (E) and late or atrial (A) diastolic flow velocities, and deceleration time of the early diastolic wave were assessed from the apical 4-chamber view using a pulsed-wave Doppler. The pulsed-wave tissue Doppler was used to evaluate peak early diastolic tissue velocity of the mitral annulus from either the lateral or septal side. Then, the ratio of E to the e’ velocity as an average value from both parts of the mitral annulus was calculated.

The assessment of LV and LA deformation was performed using the semiautomated 2-dimensional speckle-tracking technique. The global longitudinal strain (GLS) of the LV was calculated as the average value of measurements obtained from three apical views (four-chamber, three-chamber, and two-chamber). The peak value of atrial longitudinal strain measured during the LV systole was used to determine peak atrial longitudinal strain (PALS). The value of the strain at the onset of the P-wave in the electrocardiogram was measured as peak atrial contractile strain (PACS). The final values of LA strain were the averages from two apical views (four-chamber and two-chamber)

All echocardiographic parameters were averaged over 3 consecutive cardiac cycles.

### 2.5. 24 h ABPM

ABPM was conducted using a validated device, the Mobil-O-Graph (GmbH, Stolberg, Germany) in accordance with established guidelines [8]. Measurements were recorded every 20 min during the daytime and every 30 min at night [9]. Daytime and nighttime periods were determined for each participant based on self-reported sleep and wake times. All individuals were instructed to maintain their normal daily routines and to keep a diary of their activities.

### 2.6. Cardiopulmonary Exercise Testing and Exercise BP

Cardiopulmonary exercise testing on a treadmill (TMX 425 (Full Vision Drive, Newton, KS, USA)) was performed according to recommendations [10] using the Bruce protocol with standard equipment. The test was terminated after reaching volitional fatigue. Exertional BP was measured during the last two minutes of each test stage using a manually operated mercury-free sphygmomanometer (A&D UM-101B, A&D Co., Ltd., Saitama, Japan), validated previously [11]. Ventilation, oxygen uptake, and carbon dioxide production were assessed continuously. Peak oxygen uptake (peak VO_2_) was calculated as the average oxygen consumption during the last 30 s of exercise.

Based on the median peak VO_2_ value of 29.3 mL/kg/min, the study population was categorized into two groups: Group 1, consisting of individuals with peak VO_2_ below 29.3 mL/kg/min, and Group 2, comprising those with peak VO_2_ above this threshold.

### 2.7. Statistical Analysis

Continuous variables were expressed as means and standard deviations (SD) due to normal distribution and compared using the Student’s *t* test. Categorical variables were presented as counts and percentages, with comparisons conducted using the chi-squared test, incorporating Yates’ correction when appropriate. Relationships between parameters were examined using the Pearson correlation coefficient for parametric variables and the Spearman correlation coefficient for non-parametric variables. A series of stepwise multiple linear regression models were performed to identify independent determinants of peak VO_2_ as a marker of exercise capacity. The components of these models were selected based on univariable associations. The variables were placed in the models when the univariate analysis *p*-value was less than 0.2. Effect size was evaluated using the Cohen’s d method. A total of 80 participants, with 40 in each group, provided an 80% statistical power at an alpha level of 0.05 to detect a significant difference in TNF-R1.

To assess the relationship between the serum TNF-R1 concentration and exercise capacity, mediation analyses were performed to test a potential causal relationship between TNF-R1 and peak VO_2_, with markers of LV function serving as mediators. The Sobel test was used to assess whether the indirect effect of the independent variable (TNF-R1) on the dependent variable (peak VO_2_) through the mediator variable (PALS, e’) is significant.

Statistical analyses were conducted using standard statistical software (Statistica version 13, TIBCO Software Inc., Palo Alto, CA, USA). A *p*-value of less than 0.05 considered statistically significant.

## 3. Results

### 3.1. Demographic and Clinical Characteristics

The demographic, clinical, and laboratory characteristics of the study population are presented in Table 1.

Group 2, with a higher exercise capacity, was found to be significantly younger and exhibited higher exercise heart rate and exercise duration compared to Group 1. The eGFR value was significantly higher in Group 2 than in Group 1, while serum TNF-R1 concentration was significantly lower in Group 2 compared to Group 1. The difference in TNF-R1 between the groups exhibited a medium effect size (Cohen’s d = 0.58).

Both groups presented similar average values of SBP and DBP in resting measurements as well as for mean SPB in the 24 H AMBP. However, the mean value of DBP in the 24 H ABPM was found to be statistically significantly lower in Group 1 compared to Group 2. The prescription of antihypertensive medications was similar in both groups.

### 3.2. Echocardiographic Characteristics

Echocardiographic parameters in the studied groups are presented in Table 2.

No differences were noted between the groups in LV dimensions, mass index, ejection fraction, and GLS. Both groups presented similar LAVI and Doppler parameters of mitral inflow, such as E/A ratio and DT. However, Group 1 exhibited significantly higher E/e’ ratio, and lower e’ velocities and PALS than Group 2, all of which indicate poorer LV diastolic properties.

### 3.3. Independent Determinants of Peak VO_2_

Univariate associations between the studied parameters are presented in Table 3.

Significant negative correlations were demonstrated between peak VO_2_ and age, serum TNF-R1 concentration, and E/e’ ratio, as well as between serum TNF-R1 concentration and PALS and e’. Positive correlations were noted between peak VO_2_ and eGFR, PALS, and e’, as well as between serum TNF-R1 concentration and E/e’ ratio.

Serum TNF-R1 concentration was found to be an independent determinant of peak VO_2_ in each of the several multivariable regression models performed (Table 4).

**Figure 1:**
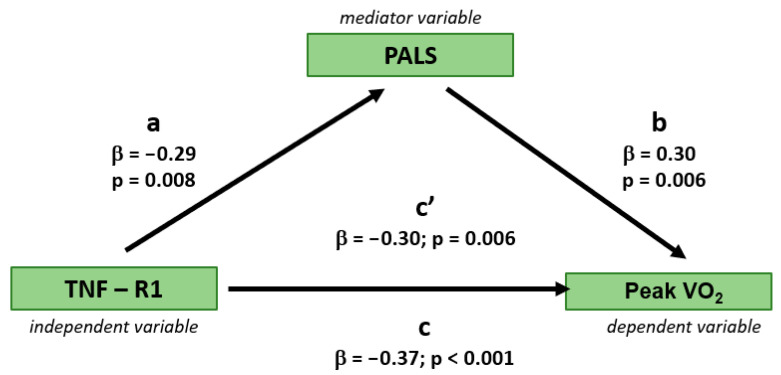
Mediating effect of peak atrial longitudinal strain (PALS) as a marker of diastolic function of the left ventricle on the relationship between serum TNF-R1 concentration on peak VO_2_ as a marker of exercise capacity. β—beta coefficient; a—beta coefficient of serum TNF-R1 concentration predicting PALS in an univariable regression analysis; b—beta coefficient of PALS predicting peak VO_2_ in an univariable regression analysis; c—beta coefficient of serum TNF-R1 concentration predicting peak VO_2_ in an univariable regression analysis without the mediating effect of PALS; c’—beta coefficient of serum TNF-R1 concentration predicting peak VO_2_ in an univariable regression analysis with the mediating effect of PALS.

**Figure 2:**
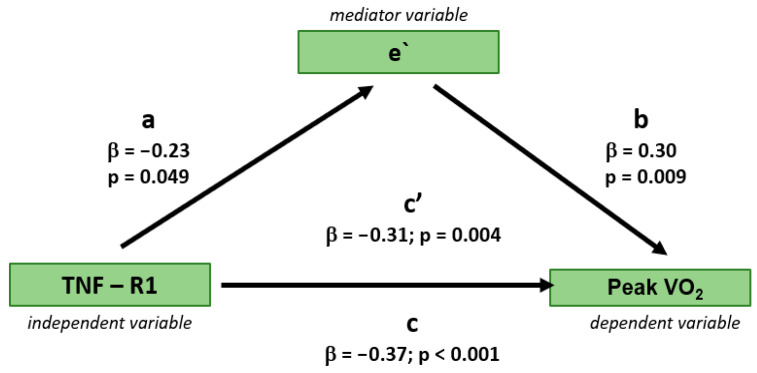
Mediating effect of early peak velocity of mitral annulus as a marker of diastolic function of left ventricle on the relationship between serum concentration of TNF-R1 on peak VO_2_ as a marker of exercise capacity. β beta coefficient; a—beta coefficient of serum TNF-R1 concentration predicting e’ in an univariable regression analysis; b—beta coefficient of predicting peak VO_2_ in an univariable regression analysis; c—beta coefficient of serum TNF-R1 concentration predicting peak VO_2_ in an univariable regression analysis without the mediating effect of e’; c’—beta coefficient of serum TNF-R1 concentration predicting peak VO_2_ in an univariable regression analysis with the mediating effect of e’.

Regression-based mediation analysis demonstrated that the relationship between serum TNF-R1 concentration and exercise capacity (peak VO_2_) was partially mediated by markers of LV diastolic function, specifically e’ and PALS, as assessed in separate models. The mediation effect was evidenced by a reduction in the variance of peak VO_2_ explained by serum TNF-R1 concentration after the inclusion of either PALS or e’ to the models, resulting in a decrease in the beta coefficient (from 0.37 to 0.30 and 0.31, respectively; Figure 1 and Figure 2). The statistical significance of the indirect effect of TNF-R1 on peak VO_2_ through mediator variables, i.e., PALS and e’, was demonstrated by the Sobel test (*p* value = 0.026 for PALS and 0.048 for e’).

The reproducibility of LV and LA strain measurements was reported previously [12,13].

## 4. Discussion

The current study demonstrates a link between the peripheral blood soluble form of serum TNF-R1 concentration and exercise capacity in patients with stable hypertensive heart disease, with higher TNF-R1 being associated with lower peak oxygen consumption. This relationship may be mediated by the deterioration of LV diastolic function, as measured by peak atrial longitudinal strain (PALS) and tissue early diastolic velocity (e’).

TNF-alpha, a proinflammatory cytokine with potent negative inotropic properties, is elevated in many cardiac conditions, including HF. It exerts its effects by binding to two receptors: TNF-R1 and TNF-R2, with the former being responsible for the cardiodepressant effects of TNF-alpha [14,15,16,17]. In experimental models of hypertension, increased BP induces a compensatory mechanism in the heart, where the LV becomes progressively thicker and less efficient in muscle relaxation and, in the later stages, in muscle contraction. Evidence from pressure overload models indicates an active role of TNF-alpha and TNF-R1 signalling in the development of hypertensive cardiomyopathy. LV hypertrophy in HHD may be accompanied by functional changes, including reduced elasticity and increased stiffness of the LV, leading initially to diastolic and longitudinal systolic dysfunction and, over time, to the decrease in the overall systolic performance [18,19].

Accumulating clinical data support the role of TNF-R1 signalling in the pathogenesis of HF progression. It has been reported that soluble TNF-R1 is a key factor mediating the relationship between comorbidities and cardiac structure and function [20,21,22,23,24]. TNF-R1 has been associated with incident HF in two independent Swedish cohorts, as well as in the U.S.-based Health ABC and Body Composition studies [25,26,27]. In the KaRen biomarker study, TNF-R1 was linked to clinical outcomes and correlated with the E/e’ ratio, a marker of diastolic dysfunction [28].

Reduced exercise capacity in HHD tends to develop gradually and precedes the onset of overt HF [29,30]. It has been found that declining exercise capacity correlates with an increased rate of progression from prehypertension to overt hypertension [31]. The underlying causes of reduced exercise capacity are multifactorial, involving metabolic, neurohormonal, proinflammatory, and profibrotic mechanisms. However, the precise pathways remain incompletely understood, and the TNF-alpha proinflammatory axis, including TNF-R1, is postulated to be involved.

The current study identified the association between higher TNF-R1 and reduced exercise capacity in the asymptomatic cohort of patients with a well-controlled hypertension, categorized as stage A or stage B HF. Despite the absence of exercise intolerance or other signs/symptoms of HF, this population might have been suspected of having different levels of physical fitness, as strong evidence exists that exercise capacity is reduced in stage B HF, i.e., in the preclinical disease with cardiac structural and/or functional derangements [32,33]. Our mediation analysis revealed that the mechanism linking TNF-R1 and peak VO_2_ might be LV diastolic dysfunction, as assessed by PALS and e’. This finding is consistent with the long-recognized contribution of LV diastolic abnormalities to the progression of exercise intolerance in HHD, but the possible role of the TNF-alpha axis in this interaction has not been clearly reported [34]. In addition to the traditional diastolic parameter, which is e’, LA strain emerged as a significant predictor of peak oxygen uptake. It has recently gained the status of a valid LV diastolic marker, being included in the updated algorithm for grading LV diastolic dysfunction [35,36,37,38,39,40]. LA strain may better represent early functional and structural remodelling, reflecting both impaired LV filling and reduced LA compliance [41]. The identification of LV diastolic function parameters as correlates of peak oxygen consumption is an expected finding and aligns with previous reports [42,43]. Subclinical LV filling abnormalities may emerge in the early stages of HHD, leading to higher pulmonary capillary pressure and a blunted increase in stroke volume under stress, resulting in reduced oxygen delivery to the muscles and, consequently, lower aerobic capacity.

Patient age and individual physical activity levels are important determinants of exercise capacity. Advancing age impairs physical performance by altering metabolic physiology and reducing both myocardial and skeletal muscle reserves. This study confirmed a significant inverse association between age and peak oxygen uptake, which paralleled the correlation observed between TNF-R1 levels and peak VO_2_. However, multivariable analysis revealed an age-independent contribution of TNF-R1 to peak VO_2_, suggesting that this signalling pathway may play a distinct role in exercise physiology.

The contribution of hypertension to the pathogenesis of HF includes promoting systemic inflammation, and inflammatory biomarkers may offer value in the diagnostic and prognostic evaluation in this condition. Assessing circulating proinflammatory cytokine levels in patients with HHD might provide valuable insights into identifying patients at risk for HF, thereby enhancing the decision-making regarding preventive interventions [44,45]. In addition to the inflammatory pathways, other TNR-R1 mediated mechanisms leading to reduced myocardial compliance and diastolic dysfunction should be considered, including oxidative stress, fibrosis, and apoptosis [6]. While our study demonstrated a relationship between elevated TNF-R1 levels, reduced exercise capacity, and impaired LV diastolic function, it is premature to postulate that targeting TNF-R1 or its downstream pathways could avert HF development. Similarly, given the exploratory nature of our study, it is too early to warrant the inclusion of TNF-R1 to existing predictive models without further validation. More research evidence is needed to consider the use TNF-R1 as a diagnostic tool in clinical practice.

### Limitations

First, the current findings are based on a relatively small group of patients in the preclinical stage of HHD. A larger sample size would strengthen the conclusions and allow for more robust statistical analysis. We consider this study to be preliminary and are planning to extend the research to a larger cohort. Second, due to the cross-sectional study design, it is impossible to establish definitive causal links based on the associations that have been identified. Third, the recruitment of patients from a single centre may limit the generalizability of the findings from this study to other populations with HHD. Fourth, since the study exclusively included Caucasian participants, caution must be exercised when applying the current results to other ethnic groups. Finally, we did not consider individual physical activity levels in our analysis, which might modify the relationship between TNF-R1 and peak VO_2_. However, due to the limited availability of precise data on this variable in our cohort, this limitation needs to be addressed in future studies.

## 5. Conclusions

In the preclinical stage of hypertensive heart disease, higher circulating levels of TNF-R1 are associated with reduced exercise capacity, and this relationship may be mediated by the decline in LV diastolic function. These findings might suggest a role of TNF signalling in the early stage of HF development, setting the stage for further studies to assess the utility of TNF-R1 in identifying individuals at risk for HF progression.

## Figures and Tables

**Table 1:** Clinical and laboratory characteristics of studied groups.

Parameter	All	Group 1 n = 40	Group 2 n = 40	*p* Value
Age, years (SD)	56.0 (13.3)	62.8 (1.7)	49.1 (11.2)	<0.001
Female, n (%)	42 (53)	26 (65)	16 (40)	0.026
BMI, kg∙m^−2^ (SD)	27.6 (4.1)	28.2 (4.8)	27.0 (3.1)	0.191
Smoking, n (%)	12 (15)	5 (13)	7 (18)	0.756
Heart rate rest, 1/min (SD)	77 (15)	76 (14)	77 (16)	0.712
Heart rate exercise, 1/min (SD)	160 (18)	153 (20)	158 (16)	0.001
SBP rest, mmHg (SD)	123 (14)	123 (15)	123 (13)	0.886
SBP exercise, mmHg (SD)	178 (25)	172 (20)	183 (28)	0.055
DBP rest, mmHg (SD)	78 (10)	78 (9)	78 (11)	0.869
DBP exercise, mmHg (SD)	83 (14)	83 (11)	84 (15)	0.904
24-Hour SBP mmHg (SD)	118 (8)	117 (9)	120 (8)	0.130
24-Hour DBP mmHg (SD)	75 (7)	72 (7)	77 (6)	0.007
NT-proBNP, ng∙mL^−1^ (SD)	4.06 (0.57)	3.99 (0.67)	4.13 (0.42)	0.390
Hemoglobin, g∙dL^−1^ (SD)	13.95 (1.0)	13.7 (1.0)	14.2 (1.0)	0.111
eGFR, mL∙min^−1^∙1.73 m^−2^ (SD)	79 (15)	74 (11.7)	85 (16)	0.008
TNF-R1, pg/mL (SD)	1303 (379)	1410 (471)	1197 (216)	0.011
Peak VO_2,_ kg/mL/min (SD)	30.0 (8.7)	22.7 (4.4)	37.3 (5.0)	<0.001
Exercise time, s (SD)	558 (220)	420 (167)	695 (179)	<0.001
**Antihypertensive treatment**				
ACEI and ARB, n (%)	49 (61)	28 (70)	21 (52)	0.169
BB, n (%)	17 (21)	10 (25)	7 (18)	0.584
CB, n (%)	26 (33)	11 (28)	15 (38)	0.177
MRA, n (%)	12 (15)	5 (13)	7 (18)	0.754
Diuretic, n (%)	14 (18)	8 (20)	6 (15)	0.694

ACEI, angiotensin-converting enzyme inhibitors; ARB, angiotensin receptor blockers; BB, beta-blocker; BMI, body mass index; CB, calcium blocker; eGFR, estimated glomerular filtration rate; DBP, diastolic blood pressure; NT-proBNP, N-terminal prohormone of brain natriuretic peptide; TNF-R1, tumour necrosis factor receptor-1; SBP, systolic blood pressure; VO_2_, oxygen uptake.

**Table 2:** Echocardiographic characteristic of studied population.

Parameter	All	Group 1 n = 40	Group 2 n = 40	*p* Value
LVDd, mm (SD)	47.7 (4,2)	48.4 (4.1)	46.9 (4.2)	0.111
LVMI g∙m^−2^ (SD)	90.0 (16.7)	92.0 (18.2)	88.0 (15.1)	0.289
LVEF, % (SD)	59 (6)	59 (7)	59 (6)	0.842
GLS, % (SD)	17.9 (3.0)	18.0 (3.4)	17.8 (2.6)	0.711
LAVI, mL∙m^−2^ (SD)	30.5 (7.3)	31.0 (8.1)	29.9 (6.5)	0.524
E/A (SD)	1.27 (0.42)	1.17 (0.36)	1.37 (0.45)	0.033
DT ms (SD)	188 (47)	191 (42)	184 (51)	0.515
e’ septal, m∙s^−1^ (SD)	0.087 (0.023)	0.080 (0.024)	0.094 (0.019)	0.007
e’ lateral, m∙s^−1^ (SD)	0.113 (0.032)	0.103 (0.031)	0.123 (0.030)	0.006
E/e’ (SD)	8.5 (3.0)	9.3 (3.4)	7.7 (2.1)	0.008
PALS, % (SD)	26.3 (6.9)	23.9 (7.4)	28.7 (5.5)	0.001
PACS, % (SD)	12.5 (4.0)	12.1 (4.6)	12.9 (3.4)	0.376

e’, early peak diastolic velocity of mitral anulus; E/e’, ratio of peak early mitral inflow to early diastolic peak velocity of mitral annulus; E/A, ratio of early to late mitral inflow velocity; DT, deceleration time; GLS, global longitudinal strain; IVDd, intraventricular end-diastolic diameter; LAVI, left atrium volume index; LVEF, ejection fraction of left ventricle; LVMI, left ventricle mass index; LVDd, left ventricular end-diastolic dimeter; PACS, peak atrial contraction strain; PALS, peak atrial longitudinal strain; PWDd, posterior wall end-diastolic diameter; SD standard deviation. 95% confidence intervals in Group 1 and Group 2 were for PALS 21.5–26.2 and 27.0–30.5, and for e’ septal 0.073–0.088 and 0.088–0.101, respectively.

**Table 3:** Univariable associations of studied parameters.

Parameter	Age		eGFR		TNF R1		PALS		E/e’		e’ Sept	
	r	*p*	R	*p*	r	*p*	r	*p*	r	*p*	r	*p*
Peak VO_2_	−0.596	<0.001	0.348	0.001	−0.366	0.001	0.304	0.006	−0.353	0.002	0.297	0.009
Age	-	-	−0.286	0.035	0.307	0.006	−0.387	<0.001	0.462	<0.001	−0.511	<0.001
eGFR			-	-	−0.040	0.773	0.242	0.075	−0.163	0.249	0.128	0.368
TNF R1					-	-	−0.291	0.009	0.207	0.071	−0.225	0.049
PALS							-	-	−0.399	<0.001	0.502	<0.001
E/e’									-	-	−0.610	<0.001

E/e’, ratio of peak early mitral inflow to early diastolic peak velocity of mitral annulus; eGFR, estimated glomerular filtration rate; NT-proBNP, N-terminal prohormone of brain natriuretic peptide; PALS, peak atrial longitudinal strain; TNF-R1, tumour necrosis factor receptor-1; VO_2_, oxygen uptake.

**Table 4:** Associations of peak VO_2_ and TNF R1 with echocardiographic and clinical parameters: multivariable analysis.

	MODEL 1 Peak VO_2_ R^2^ = 0.42			MODEL 2 Peak VO_2_ R^2^ = 0.28			MODEL 3 Peak VO_2_ R^2^ = 0.25			MODEL 4 Peak VO_2_ R^2^ = 0.24			MODEL 5 Peak VO_2_ R^2^ = 0.18			MODEL 6 Peak VO_2_ R^2^ = 0.18		
	β	SE	*p*	Β	SE	*p*	β	SE	*p*	β	SE	*p*	β	SE	*p*	β	SE	*p*
Age,	−0.49	0.09	<0.001															
TNF-R1	−0.21	0.09	0.025	−0.31	0.10	0.003	−0.31	0.13	0.017	−0.31	0.11	0.004	−0.31	0.11	0.004	−0.30	0.11	0.006
eGFR	0.18	0.09	0.046	0.25	0.10	0.012	0.31	0.12	0.015	0.26	0.10	0.013						
E/e’				−0.25	0.10	0.016												
e’							0.20	0.10	0.058				0.22	0.11	0.037			
PALS										0.16	0.11	0.131				0.22	0.11	0.049

e’, early diastolic peak velocity of mitral anulus; E/e’, ratio of peak early mitral inflow to early diastolic peak velocity of mitral annulus; eGFR, estimated glomerular filtration rate; NT-proBNP, N-terminal prohormone of brain natriuretic peptide; PALS, peak atrial longitudinal strain; TNF-R1, tumour necrosis factor receptor-1; VO_2_, oxygen uptake.

## Data Availability

The original contributions presented in this study are included in the article. Further inquiries can be directed to the corresponding author(s).

## Abbreviations

The following abbreviations are used in this manuscript:

ABPM	Ambulatory Blood Pressure Monitoring
eGFR	Estimated Glomerular Filtration Rate
HHD	Hypertensive Heart Disease
HF	Heart Failure
HFpEF	Herat Failure with preserved Ejection Fraction
HFrEF	Herat Failure with reduced Ejection Fraction
GLS	Global Longitudinal Strain
LVEF	Left Ventricle Ejection Fraction
LAVI	Left Atrial Volume Index
NT-proBNP	N-terminal prohormone of brain natriuretic peptide
PACS	Peak Atrial Contraction Strain
PALS	Peak Atrial Longitudinal Strain
Peak VO_2_	Peak Oxygen Uptake
TNF-R1	Tumour necrosis factor receptor-1
